# Measurement of pharyngeal sensory cortical processing: technique and physiologic implications

**DOI:** 10.1186/1471-2202-10-76

**Published:** 2009-07-14

**Authors:** Inga K Teismann, Olaf Steinstraeter, Tobias Warnecke, E Bernd Ringelstein, Christo Pantev, Rainer Dziewas

**Affiliations:** 1 Institute for Biomagnetism and Biosignalanalysis, University of Muenster, Malmedyweg 15, 48149 Muenster, Germany; 2 Department of Neurology, University of Muenster, Albert-Schweitzer-Str.33, 48149 Muenster, Germany

## Abstract

**Background:**

Dysphagia is a major complication of different diseases affecting both the central and peripheral nervous system. Pharyngeal sensory impairment is one of the main features of neurogenic dysphagia. Therefore an objective technique to examine the cortical processing of pharyngeal sensory input would be a helpful diagnostic tool in this context. We developed a simple paradigm to perform pneumatic stimulation to both sides of the pharyngeal wall. Whole-head MEG was employed to study changes in cortical activation during this pharyngeal stimulation in nine healthy subjects. Data were analyzed by means of synthetic aperture magnetometry (SAM) and the group analysis of individual SAM data was performed using a permutation test.

**Results:**

Our results revealed bilateral activation of the caudolateral primary somatosensory cortex following sensory pharyngeal stimulation with a slight lateralization to the side of stimulation.

**Conclusion:**

The method introduced here is simple and easy to perform and might be applicable in the clinical setting. The results are in keeping with previous findings showing bihemispheric involvement in the complex task of sensory pharyngeal processing. They might also explain changes in deglutition after hemispheric strokes. The ipsilaterally lateralized processing is surprising and needs further investigation.

## Background

Dysphagia is a common complication of a variety of neurological diseases affecting both the peripheral and central nervous system. Thus, dysphagia has been reported in neurologic patients suffering from ischemic stroke, amyotrophic lateral sclerosis, Kennedy disease, myasthenia gravis and Parkinson disease as well as ENT patients with tumors or due to postoperative lesions. The consequences of oropharyngeal dysphagia can be severe: dehydration, malnutrition, aspiration, choking, pneumonia, and death. Nursing home occupants with oropharyngeal dysphagia and aspiration have 45% 12-month mortality [1].

Processing of oropharyngeal sensory information is crucial to assure safe deglutition. Impairments of sensation, as seen in stroke patients, causes severe swallowing problems [2-6]. Even induced oropharyngeal anaesthesia is known to result in short-term dysphagia in healthy subjects [7-9].

During the last years, the interest in swallowing, dysphagia and oropharyngeal sensory processing has been constantly growing. Several studies have examined the cortical activation of human swallowing [10-20] finding bilateral processing in the primary somatosensory cortex during swallowing execution. Reduced sensory input, due to local oropharyngeal anesthesia, leads to significantly reduced bilateral cortical activation during swallowing processing in healthy subjects [21].

Little is known about cortical processing of sensory pharyngeal stimulation. In the present study we examined nine healthy subjects by means of magnetoencephalography (MEG) and synthetic aperture methods (SAM). In each subject both sides of the pharyngeal wall were stimulated. We hypothesized bilateral activation of the lateral sensorimotor cortex following sensory pharyngeal stimulation.

## Results

All participants tolerated tube placement and air puff application without any difficulties. No coughing and especially no signs of aspiration occurred during tube placement and measurements. No swallowing was elicited by the pneumatic stimulation. None of the subjects complained about an urge to swallow due to the stimulation. Subjects were instructed to swallow between the air pulses. Therefore we tried to reduce movement artifacts in the examined time interval. Localization of the stimulation area was depending on the statement of the subjects. The position of the tube was adjusted until each subject stated sensory sensation at the lateral pharyngeal wall corresponding to the chosen nostril. During and after each MEG recording session subjects had to state whether the stimulation area was unchanged. If the tube position changed during recording, the whole measurement was repeated. This problem occurred only once.

**Figure 1:**
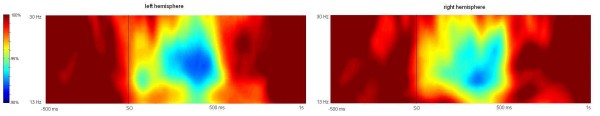
**Time-frequency wavelet plots**. Wavelet analysis of the virtual channels representing the individual maximum ERD (left: maximum ERD over the left hemisphere; right: maximum ERD over the right hemisphere). Colors represent the percental change of frequency power relative to baseline (100%) as indicated in the color bar. Changes relative to the baseline interval (-0.5 – 0 sec., whereas 0 is SO) are calculated separately for each frequency. The time points used to define the time intervals of interest are marked (SO = stimulus onset). A distinct decrease of frequency power in the beta frequency range after stimulus onset and a re-increase after pneumatic stimulation stopped is found. The effect is comparable in both hemispheres.

Wavelet analysis of virtual channel recordings over the individual maximum event related desynchronization (ERD) in each hemisphere revealed a reduction of power in the beta frequency range directly after stimulus onset. A re-increase of power was found after ending of sensory stimulation. ANOVA and *post-hoc *t-tests revealed a decrease of beta power from the 'control time window' to the 'active time window' after stimulation to both sides of the pharyngeal wall and in both hemispheres [see figure 1].

According to these time-frequency plots SAM analysis was calculated for the two relevant frequency bands, alpha and beta comparing the 'active time window' to the 'control time window'.

Individual SAM analysis of the alpha and beta frequency band resulted in bilateral ERD within the caudolateral primary somatosensory cortex for both stimulation sides in all subjects.

**Figure 2:**
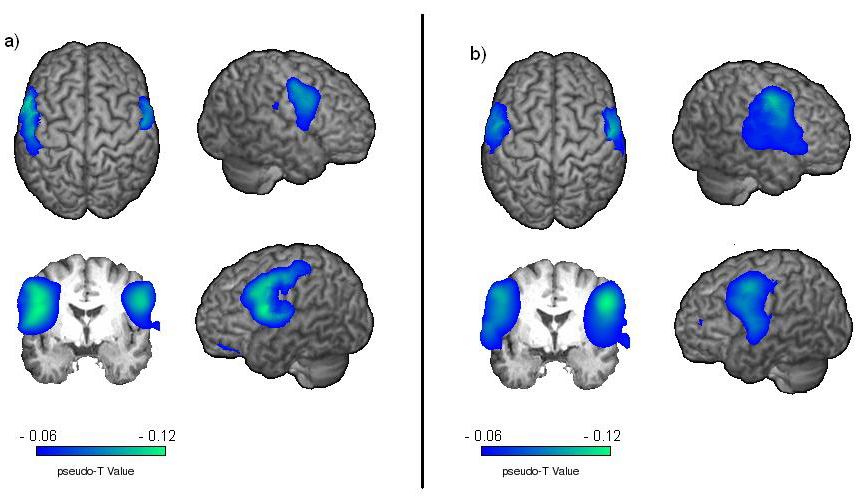
**Event related desynchronization**. Significant activation in group analysis is shown (p < 0.05). Changes in the beta-frequency-band during pneumatic pharyngeal activation compared to the resting stage. a) ERD evoked by pneumatic stimulation to the left side of the pharyngeal wall. Stronger and broader activation is found in the temporal lobe of the left hemisphere. b) ERD evoked by pneumatic stimulation of the right side of the pharyngeal wall. Here a right hemispheric lateralization of temporal activation can be seen. The color bar represents the t-value.

SAM group analysis resulted in significant beta ERD for both stimulated sides (p < 0.05). Again maximum ERD were located bilaterally within the caudolateral primary somatosensory cortex, corresponding to Brodmann areas (BA) 1, 2, and 3 but also spread into the motor cortex and secondary somatosensory areas (BA 4, 6, 5, and 40) [see table 1 and figure 2]. No significant activation in group analysis was observed in any other cortical area or in the alpha frequency range.

**Table 1:** Talairach coordinates and the corresponding Brodmann area of task-related activation across the two different stimulation sides in both hemispheres are shown.

Stimulation	Right	Right	Left	Left
Hemisphere	Right	Left	Right	Right

Talairach Coordinate	62.37, -10.43, 24.47	-57.42, -14.91, 32.07	63.36, -9.55, 22.59	-54.45, -10.43, 43.82

Brodmann Area	3	3	3	3

Regarding hemispheric lateralization stronger activation was observed in the ipsilateral hemisphere side. Stimulation of the left pharyngeal wall resulted in a left lateralized cortical activation (LI: 0.61), while a reverse, but much weaker effect was seen in right sided stimulation (LI: -0.16).

## Discussion

This is one of the first studies presenting a simple and easy to perform paradigm to examine the cortical processing of sensory pharyngeal stimulation by means of MEG. The pneumatic stimulation of the pharyngeal wall was well tolerated by all subjects and led to bilateral activation of the caudolateral primary somatosensory cortex. The exact stimulation location cannot be detected. Therefore we were only depending on the statement of the subjects. A bilateral stimulation due to malpositioning or tube movement cannot definitely be excluded.

Some previous MEG and EEG studies found changes in the central alpha rhythm linked to the somatosensory system, while beta changes are linked to the motor system [22]. In contrast to this significant activation was only found in the beta frequency range in the present study.

The activated somatosensory cortex is part of the facial area and known to process afferent pharyngeal information since Brodmann [23]. In a MEG study electrical stimulation of the pharynx elicited activation in the same area of the primary somatosensory cortex [24]. The localization has also been confirmed by positron emission tomography [12] and in epilepsy surgery [25]. Also two recent fMRI studies examining pneumatic stimulation of the oropharynx found bilateral activation of the sensorimotor cortex [26,27].

While the relevance of sensory information for secure deglutition is beyond controversy, a specific examination of its cortical processing has rarely been performed. An increased cortical excitability after electrical pharyngeal stimulation and a somatotopic cortical representation of swallowing related musculature could be shown by transcranial magnetic stimulation [28]. Up to now, examinations of the neuronal activation after pharyngeal stimulation mainly addressed therapeutic options for dysphagic patients. Transcranial magnetic stimulation studies demonstrated that peripheral intraluminal low-frequency stimulation in humans resulted in increased corticobulbar excitability in health and after stroke [29,30]. Other studies focused on the influence of pharyngeal stimulation on deglutition. Cold and taste stimulation of the faucial area hastens the onset of the pharyngeal swallowing phase and reduces swallowing latency [31,32]. Electrical stimulation of the superior laryngeal nerve in cats resulted in reduced swallowing latency [33].

The second and more surprising finding of this study was a slight ipsilateral lateralized cortical processing following pharyngeal stimulation. Afferent information of the oropharynx is transferred by the glossopharngeal nerve to the nucleus spinalis nervi trigemini located in the brain stem. After crossing the midline it ascends to the thalamus and afterwards reaches the primary somatosensory cortex [34,35]. Decussation of sensory as well as motor fibres is one of the main principles of the central nervous system. Altogether about 90% of these fibres cross over to the contralateral side in the mesencephalon. This leads to a strong contralateral lateralization of most cerebral processes which have been demonstrated frequently for the auditory, visual, sensory and motor system. Only for the olfactory system a mainly ipsilateral cortical processing has been shown [36]. Therefore the bilateral and even slight ipsilateral lateralized processing found in our MEG data is quite remarkable. A similar effect has previously been shown after repetitive transcranial magnetic stimulation of the pharyngeal motor cortex. Here a visible stronger and longer ipsilateral activation was seen. Nevertheless this observation did not reach significance during further calculations [37]. In an fMRI study oral stimulation of only the right side resulted in bilateral cortical activation [27]. Our results are also supported by previous findings showing bilateral hemispheric involvement in the complex task of sensory pharyngeal processing in a PET study [12]. Bihemispheric involvement is also reflected in patients with hemispheric stroke. Both right and left hemispheric strokes can lead to sensory impairment of the oropharyngeal area resulting in severe deglutition [2,38,39]. It also correlates with a bilateral motor activation seen in reflexive swallowing [10]. A recent fMRI study found bilateral sensorimotor activation with a slightly contralateral lateralization after oropharyngeal stimulation [26]. Apart from methodological differences the stimulated areas varied from those in our study. Sörös et al. focused on the oral part of the pharyngeal cavity and therefore stimulation was delivered through a dental splint. Contrary to this the stimulation in our study was performed using a nasal tube and delivered to the lower part of the pharynx. The difference of stimulation locality might explain the reverse lateralization in cortical activation.

We therefore conclude that the anatomical decussation of the pharyngeal afferences does not lead to a functional correlate. The ipsilateral hemisphere seems to be at least as involved in sensory oropharyngeal input as the contralateral one.

The pneumatic stimulation paradigm is able to indicate the corresponding cortical areas processing pharyngeal sensor information. This easy and quite non invasive measurement is objective and can also be performed on different groups of dysphagic patients to increase our understanding about the physiology and pathophysiology of pharyngeal sensor processing. It might also be helpful to monitor swallowing recovery after stroke as well as other neurogenic or morphologic alterations leading to dysphagia or even to observe and compare different therapies.

## Conclusion

In conclusion the new stimulation method introduced here is in principle simple and easy to perform and might therefore also be applicable in the clinical setting. The results reveal bilateral hemispheric involvement in the complex task of sensory pharyngeal processing. They might also explain frequently changes in deglutition after hemispheric strokes, showing likewise disturbances after right as well as left hemispheric stroke. Additionally, the slight ipsilaterally lateralized processing is surprising and needs further investigation.

## Methods

### Subjects

Nine healthy right-handed volunteers (5 males, age range 23 – 35 years, mean 29 years) served as subjects. The local regional ethics committee approved the protocol of the study. Informed consent was obtained from each subject after the nature of the study was explained in accordance to the principles of the Declaration of Helsinki.

### Sensory pharyngeal stimulation

**Figure 3:**
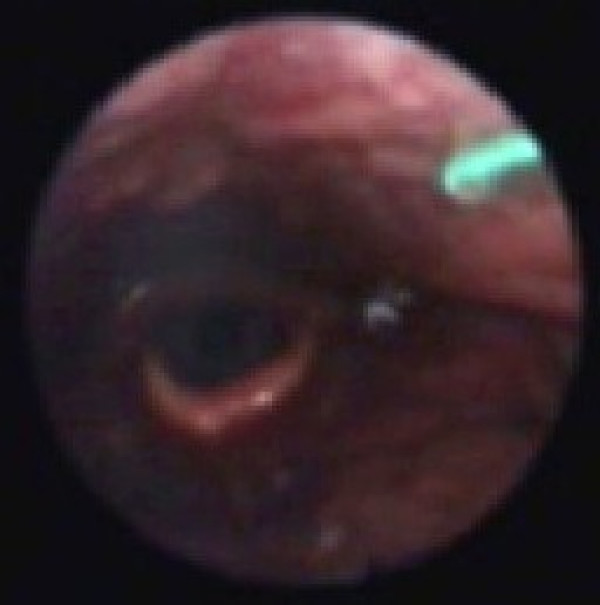
**Position of the nasogastric tube**. The endoscopic picture illustrates the position of the gastric tube during pneumatic stimulation in one of the subjects. In this picture the tip of the tube is placed through the left nostril.

A baby gastric tube (Unomedical, Sterile EO, 40 cm length, 1 1/3 mm diameter) was used for pneumatic stimulation. Air puffs were administered by compressed oxygen with a flow of 2 l/min through the tube. One of the two lateral apertures of the gastric tube was sealed and the tube was placed through one nostril and advanced forward until visible under the velum. The position of the tube tip was adjusted until the subject stated to feel the air puffs laterally on the pharyngeal wall at the side of the nostril the tube was placed in [see figure 3].

A 24 minute measurement was performed with the air puffs being applied with a stimulation frequency of 0.5 Hz and 500 ms stimulus duration. Stimulus duration was chosen to gain a reasonable long time interval for SAM analyses. The inter-stimulus interval in MEG studies with somatosensory stimulation is varying between 0.33 and 3 seconds in the actual literature [40-44]. In the present study the stimulus frequency of 0.5 Hz was chosen to keep the measurement duration short on the one hand and gaining sufficient recovery of the active nerve cells on the other hand. A 5 liter bottle of oxygen was connected to a pneumatic stimulation device. During stimulation an oxygen flow of 2 l/min was chosen.

### MEG recording

A whole head 275-channel SQUID sensor array (Omega 275, CTF Systems Inc.) was used to collect MEG data with a sample frequency of 600 Hz. Data was filtered during acquisition using a 150 Hz low-pass filter. Subjects were seated in a comfortably upright position watching a self selected silent movie.

### Anatomical MRI

MRI data were acquired on a 3.0 T Scanner (Gyroscan Intera, Philips) with a standard head coil. T1-weighted sagittal anatomical images with in-plane resolution of 512 × 512 (0.6 × 0.6 mm resolution) and 320 slices (0.5 mm thickness) were recorded using spoiled gradient echo imaging.

### Data analysis

To define the active frequency bands time-frequency plots were calculated using wavelet analysis. These calculations were done using EMEGS (ElectroMagnetic-EncephaloGraphy Software; ), a tool for analyzing neuroscientific data developed in MATLAB [45]. The 275 channels of the MEG system were fragmented into 10 channel groups, frontal, central, parietal, temporal and occipital channels in each hemisphere. Data from each individual subject was averaged across trials (-0.8 to 1.2 s in reference to stimulus onset) and time-frequency analysis was performed (0 – 150 Hz). The time-frequency plots of the parietal channels were determined for both hemispheres and averaged across all subjects in each group.

According to the changes of the time-frequency analysis MEG data were than filtered within two frequency bands: alpha (8–13 Hz) and beta (13–30 Hz). SAM was used to generate a 20 × 20 × 14 cm^3 ^volumetric pseudo-t images [46] from the filtered MEG signals, with 3-mm voxel resolution. A pseudo-t value cancels the common-mode brain activity by subtracting the source power found in a defined control stage from the source power in the active stage. To account for uncorrelated sensor noise, this difference is normalized by the mapped noise power [46,47]. For analyzing cortical activity during "active time window" (0 – 0.5) the corresponding "control time window" (-0.5 – 0) served as control.

Group analysis of multiple subjects' data was performed as previously published [22,48-50]. Briefly, the individual MRIs were first transformed into a common anatomical space using SPM2. Then the spatial normalized activation maps were obtained by applying this transformation to the individual SAM volumes. The significance of activated brain regions was investigated by the permutation test for the observed time interval. To calculate difference between stimulation sides a standard permutation test for paired samples was performed [51].

Hemispheric lateralization of brain activation was quantified using a lateralization index (LI), which was calculated as (L-R)/(L+R), where L and R are the cumulative pseudo-t activation in the somatosensory cortex (BA 3, 1 and 2, according to the Talairach atlas) of the left and right hemisphere, respectively. A positive LI indicates left hemispheric lateralization, while a negative LI indicates stronger right hemispheric activation. Ratios around 0 represent indeterminate dominance, 1, respectively -1 are indicating unilateral activation [10,52].

To examine the temporal sequencing of activation virtual channels were calculated individually for the maximum beta ERD in each hemisphere. Afterwards time-frequency plots of virtual channel activation were calculated and grand averaged using EMEGS. Afterwards the two time intervals "active time window" (-0.5 – 0) and "control time window" (0 – 0.5) were defined for further calculations. Comparisons between different stimulation sides, time intervals and hemispheres were performed using two-way ANOVA followed by *post-hoc *t-tests.

## Authors' contributions

IT performed analysis and interpretation of data and drafted the manuscript. She was funded by the Deutsche Forschungsgemeinschaft. OS has made analysis and interpretation of data and was involved in drafting the manuscript. TW helped in the development of the paradigm and performed several of the measurements. CP and EBR revised the manuscript critically for important intellectual content. RD made substantial contributions to conception and design, and has given final approval of the version to be published. All authors read and approved the final manuscript
